# Which Virus Will Cause the Next Pandemic?

**DOI:** 10.3390/v15010199

**Published:** 2023-01-10

**Authors:** Gabriele Neumann, Yoshihiro Kawaoka

**Affiliations:** 1 Department of Pathobiological Sciences, University of Wisconsin-Madison, Madison, WI 53711, USA; 2 Department of Virology, Institute of Medical Science, The University of Tokyo, Tokyo 108-8639, Japan; 3 International Research Center for Infectious Diseases, Institute of Medical Science, The University of Tokyo, Tokyo 108-8639, Japan; 4 Pandemic Preparedness, Infection and Advanced Research Center, The University of Tokyo, Tokyo 108-8639, Japan

**Keywords:** pandemics, viruses

## Abstract

One of the most pressing and consequential problems in infectious disease research is to better understand the potential of viruses to cause a pandemic, or, in simple terms, determine which virus will cause the next pandemic. We here define pandemics as WHO-declared pandemics, or disease outbreaks commonly referred to as pandemics that predate the WHO pandemic framework. Despite extensive research in the field of infectious diseases in recent decades, all pandemics have found us unprepared, with enormous losses of human lives, tremendous costs for public health, and vast and potentially long-lasting economic losses. Here, we discuss viruses that may cause a pandemic in the future.

## 1. Past Pandemics

In assessing the potential of infectious agents to cause a pandemic, important lessons may be learned from past pandemics. We therefore briefly review pandemics since the beginning of the 20th century, for which robust documentation is available and the causative agents have been identified.

The influenza A/H1N1 pandemic of 1918/1919: The ‘Spanish Flu’ in 1918–1919 claimed an estimated 50 million lives worldwide (Table 1). It was caused by an influenza A virus of the H1N1 subtype that most likely transmitted to humans from the animal reservoir of influenza A viruses. It spread around the globe rapidly, most likely fueled by troop movement and poor hygiene during World War I. At this time, influenza viruses had not been discovered, but modern technologies allowed the recreation of the pandemic virus from autopsy samples or frozen corpses from victims of the pandemic [1,2]. Several studies demonstrated that the viral surface protein, hemagglutinin (HA) [3,4,5,6,7,8], the polymerase proteins, particularly PB1 [4,9,10,11], the interferon-antagonist protein (NS1) [12,13], and the PB1-F2 protein [14] contributed to its virulence, but the mechanisms that would explain the severity of infections with this virus are not fully understood.

The influenza A/H2N2 pandemic: The ‘Asian’ pandemic of 1957 was caused by a reassortant influenza A virus that possessed H2 hemagglutinin (HA; the major viral antigen), N2 neuraminidase (NA; minor viral antigen), and basic polymerase 1 (PB1 genes derived from an avian influenza virus, while the remaining five viral genes were derived from previously circulating H1N1 viruses [15,16]. Humans at the time lacked immunity to influenza A viruses of the H2N2 subtype, resulting in worldwide spread of the virus and replacement of the formerly circulating A/H1N1 viruses. An estimated one million deaths worldwide were attributed to this pandemic (Table 1).

The influenza A/H3N2 pandemic of 1968: Like the pandemic of 1957, the ‘Hong Kong’ pandemic of 1968 resulted from reassortment between human and avian influenza A viruses. H3, HA, and PB1 genes of avian influenza A virus origin reassorted with the remaining segments of the human H2N2 viruses circulating at the time [15,17]. The resulting H3N2 reassortant virus encountered an immunologically naïve population that lacked antibodies to H3 HA, enabling worldwide spread and the supersession of human H2N2 viruses. The estimated death toll from this pandemic reached about 1 million (Table 1).

The reemergence of A/H1N1 viruses in 1977: In 1977, widespread influenza outbreaks were caused by an A/H1N1 influenza virus closely related to strains circulating in the early 1950s, suggesting accidental virus release or a vaccine trial with an insufficiently attenuated live virus. The reemerged A/H1N1 virus did not replace the previously circulating A/H3N2 viruses; instead, both virus subtypes co-circulated for subsequent decades (Table 1).

The influenza A/H1N1 pandemic in 2009: A triple reassortant influenza A virus possessing genes from human, avian, and swine influenza A viruses caused the 2009 pandemic [18,19,20,21] with an estimated 284,000 deaths worldwide (Table 1). The 2009 A/H1N1 viruses displaced the circulating A/H1N1 viruses, but not the co-circulating A/H3N2 viruses. Influenza A/H1N1 viruses had been circulating in humans for several decades, so A/H1N1 viruses were not considered a pandemic threat. However, the 2009 pandemic H1N1 virus was sufficiently antigenically different from the previously circulating A/H1N1 viruses to evade immunity and cause a pandemic.

The SARS-CoV-2 pandemic: In late 2019, reports of an unknown respiratory disease emerged [22] and in early 2020, the causative agent was identified as a novel coronavirus, later termed SARS-CoV-2 (severe acute respiratory syndrome coronavirus 2) [23]. The illness caused by SARS-CoV-2 was termed COVID-19 (coronavirus disease 19). By late August, 2022, more than 600 million cases and approximately 6.5 million deaths had been reported (Table 1). The economic costs are in the trillions and the socioeconomical impact has exceeded anything most people have ever experienced.

## 2. Public Health Emergencies of International Concern (PHEIC) and WHO Priority Diseases

In 2005, the WHO established the International Health Regulations (IHR), a formal framework for countries to promptly respond to Public Health Emergencies of International Concern (PHEIC), defined as “an extraordinary event which is determined to constitute a public health risk to other States through the international spread of disease and to potentially require a coordinated international response”. Four questions guide the WHO member states in deciding if the WHO should be informed of a potential PHEIC: (i) Is the public health impact of the event serious?; (ii) Is the event unusual or unexpected?; (iii) Is there a significant risk for international spread?; or (iv) Is there a significant risk for international travel or trade restrictions? If at least two of these criteria apply, a member state should immediately notify the WHO so that the IHR Emergency Committee can vote on declaring a PHEIC. Additionally, occurrences of smallpox, wild-type poliomyelitis, SARS (severe acute respiratory syndrome), and any new subtype of human influenza are automatically a PHEIC [24].

Since 2005, seven PHEICs have been declared, all of them viral emerging infectious diseases:

*(i)*  2009 H1N1 pandemic (see Past Pandemics);
*(ii)*  Ebola virus disease outbreak in Western Africa, 2013–2016: This outbreak claimed more than 11,000 lives (Table 1) and caused tremendous socioeconomical disruption in Western Africa;
*(iii)*  Poliomyelitis (since 2014): With extensive vaccination campaigns over the last decades, many countries have been declared polio virus-free. Although only a few cases had been reported in previous years, the international spread of wild-type polio virus by adult travelers prompted the IHR Emergence Committee to declare poliomyelitis a PHEIC in 2014 (Table 1);
*(iv)*  Zika virus disease outbreak in South America (2016): About 600 cases of microcephaly in newborns were linked to maternal Zika virus infections (Table 1), causing international concerns over Zika virus infections in South America;
*(v)*  Ebola virus disease outbreak in the Democratic Republic of The Congo (2018–2020): This outbreak claimed about 2300 lives (Table 1). A vaccine not yet available in 2013–2015 helped control the outbreak.
*(vi)*  SARS-CoV-2 pandemic (see Past Pandemics).
*(vii)*  Mpox outbreak: On 23 July 2022, the Mpox outbreak was declared a PHEIC based on more than 16,000 reported cases from 75 countries and territories.

The WHO also developed a list of priority diseases that “pose the greatest public health risk due to their epidemic potential and/or whether there is no or insufficient countermeasures” ([25]; accessed on 31 August 2022) (Table 1). The current list includes COVID-19, Crimean-Congo hemorrhagic fever, Ebola virus disease, Marburg virus disease, Lassa fever, SARS, MERS (Middle-East respiratory syndrome), Nipah and henipaviral disease, Rift Valley fever, Zika, and “Disease X” (i.e., a currently unknown pathogen). Interestingly, the original list in 2017 included human coronaviruses, which have now been replaced with SARS-CoV-2. In addition to the viruses and diseases already discussed, the list primarily includes zoonotic pathogens that have not (yet) caused widespread infections among humans, but may acquire this ability in the future.

## 3. What Are the Characteristics of Pandemic Viruses, PHEIC Agents, and Viruses Causing WHO Priority Diseases?

By comparing pandemic viruses, PHEIC agents, and viruses causing WHO priority diseases, important commonalities or differences that can be used to assess pandemic risk may be revealed.

Zoonoses, vector-borne diseases, and animal reservoirs: Viruses already circulating in humans are unlikely to cause a pandemic due to preexisting immunity in human populations. It is therefore much more likely that the next pandemic will be caused by a zoonotic event in which an infectious agent is transmitted from its animal reservoir to humans. In fact, almost all recorded pandemics, PHEIC events, and WHO priority diseases have been/are zoonoses (Table 1) in which whole viruses or genetic components of viruses encoding major viral antigens originated from an animal reservoir. The two notable exceptions are smallpox and polio viruses, for which humans are the only known reservoir; consequently, virus eradication has been accomplished for the smallpox virus and is within reach for the polio virus. For the other viruses discussed here, eradication is currently not feasible because their respective animal reservoirs cannot be controlled. Moreover, the number of zoonoses has greatly increased in recent decades due to habitat encroachment with more frequent contacts between humans and animals, wildlife trade, and extensive animal husbandry, among other factors.

All influenza virus pandemics have originated from wholly avian influenza viruses or influenza viruses possessing HA (and other gene segments) originating from an avian influenza virus. The natural reservoirs of avian influenza viruses are wild waterfowl, a reservoir that cannot be controlled. Reassortment of genes from avian, human, and swine influenza viruses may have occurred in poultry or pigs (for the 2009 A/H1N1 influenza pandemic).

Bats have emerged as another major reservoir of viruses that pose a risk to humans. Bats are genetically very diverse mammals that exist on all continents except for Antarctica. Thousands of different viruses have been isolated from bats from at least 28 virus families, most frequently *Paramyxoviridae*, *Coronaviridae*, and *Rhabdoviridae* [26]. Importantly, many human pathogens (including Marburg virus, Nipah virus, and Hendra virus) and the viral genomic material of some Ebolaviruses have been detected in bats, and increasing evidence points towards bats as the animal reservoir of SARS, SARS-CoV-2, MERS, and Ebola viruses (Table 1).

Arthropods are another virus reservoir of concern. Arthropod-transmitted viruses have not caused pandemics in humans, but several WHO priority diseases (including Zika virus infections, Crimean-Congo hemorrhagic fever, and Rift Valley fever) are transmitted by arthropods (Table 1). Additional zoonotic viruses of concern (including West Nile virus, yellow fever virus, dengue virus, and chikungunya virus) are also transmitted by infected mosquitos or ticks.

Viruses maintained in rodents have not been the source of viral pandemic or PHEIC events, but rodents harbor Lassa virus (which causes a WHO priority disease; Table 1) and many other viruses, primarily from the order *Bunyavirales* and the *Flaviviridae* family. Among them are several hemorrhagic fever viruses; therefore, rodents should not be discounted as reservoirs of future zoonotic viruses of high public health significance.

Immunity in humans: After virus transmission from an animal reservoir, fulminant virus spread in humans will occur only in immunologically naïve populations. Viruses that are antigenically similar to those currently circulating in humans are therefore extremely unlikely to cause a pandemic. However, immunity against viruses endemic in humans is narrow and may wane over time. Accordingly, the A/H1N1 viruses circulating in humans until 2009 did not protect against infection with the 2009 A/H1N1 pandemic influenza viruses, and exposure to human seasonal coronaviruses did not protect against SARS-CoV-2. Human immunity is also shaped by virus replacement events and discontinuation of vaccination. Influenza A viruses of the H2N2 subtype caused a pandemic in 1957 and were replaced by a novel pandemic virus in 1968. Since then, A/H2N2 viruses have not circulated in humans, leaving people born after 1968 (i.e., most of the world population) vulnerable to infection with A/H2N2 viruses. Similarly, vaccination against polio virus was discontinued in many countries around 2000, resulting in an increasing proportion of people without immune protection from this virus.

Vaccination remains the best protective option against infectious disease. Vaccines to human influenza viruses are available, but they are strain-specific and not expected to provide protection against antigenically different influenza viruses (Table 1). Vaccines against SARS-CoV-2 are now available worldwide but their efficacy may be reduced for newly emerging SARS-CoV-2 variants. Several African countries have now approved a vaccine against *Zaire ebolavirus* (Ervebo, manufactured by Merck), which may quench future *Zaire ebolavirus* outbreaks. For other PHEICs and viruses causing WHO priority diseases, vaccines are in different stages of development. The ‘American Pandemic Preparedness: Transforming our Capabilities’ plan lists the design, testing, and safety review of a vaccine within 100 days after the recognition of a pandemic threat as a major goal [27]. While this timeline was previously utopic, it may be achievable with mRNA vaccines. However, even if this goal were to be accomplished, vaccine development, mass production, and mass vaccination would take too long to prevent the first waves of novel pandemics.

Modes of human infection and human-to-human transmission: Viruses can transmit via the air, vehicles (including food, water, body fluids, and fomites), or vectors (such as mosquitoes, ticks, and fleas) (Table 1). All recorded pandemics have been caused by viruses that transmit among humans through the air. In contrast, some PHEIC events and WHO priority diseases are caused by viruses that infect humans through vectors (i.e., Zika virus and Crimean-Congo hemorrhagic fever virus) or through close contact with or the consumption of infected animals (for example, Ebola virus, Marburg virus, or Nipah virus), and transmit among humans through direct contact with body fluids (Table 1). Virus transmission via the airborne route can only be curbed through extremely strict public health measures (such as quarantines, cancellations of mass gatherings, social distancing, and mask wearing). The SARS-CoV-2 pandemic highlighted the challenges of implementing and maintaining such measures due to their socioeconomic disruption. In contrast, virus transmission through vehicles can largely be prevented through strict hygiene measures, although the Ebola virus disease outbreak in West Africa demonstrated that the implementation of such measures can be challenging if they interfere with local customs (such as burial practices).

Other factors: Other factors, such as viruses’ ability to bind to and replicate in human cells, and counteract host innate immune responses, are important for fulminant virus spread among humans. The human receptors used for virus entry are diverse and have been identified for only a few viruses. Similarly, we lack a thorough understanding of the molecular features that enable virus replication in humans. Viruses that cause disease in humans often encode interferon antagonists, which dampen the host cell responses to infection, but our data are limited to a relatively small number of viruses. While much has been learned about the molecular virology of some viruses, we cannot predict the risk potential of newly emerging viruses simply by assessing their sequence. However, it is interesting to note that zoonotic viruses are more often RNA than DNA viruses.

## 4. What Will Cause the Next Pandemic?

The next pandemic will likely result from a zoonotic event caused by a virus introduced into humans from mammals including bats (which harbor the highest proportion of zoonotic viruses among mammals) [28] and rodents, or from avian species. While Table 1 lists past and current pandemic viruses, PHEIC agents, and WHO priority diseases, it is also conceivable that the next pandemic will be caused by another zoonotic virus, such as yellow fever or the chikungunya virus. However, we suggest that a pandemic virus will have to transmit among humans via the air, which reduces the number of candidate PHEIC agents and WHO priority disease-causing viruses with pandemic potential.

Could the next pandemic be caused by a close relative of viruses endemic in humans? Most respiratory infections in humans are caused by respiratory syncytial virus, rhinoviruses, coronaviruses, parainfluenza viruses, influenza viruses, metapneumoviruses, adenoviruses, measles virus, mumps virus, enteroviruses, bocaviruses, and parvoviruses (Table 2). Except for influenza, measles, and mumps viruses, vaccines are not available for these viruses (Table 2). Close relatives of these viruses circulating in non-human reservoirs could infect humans and acquire the ability to replicate in humans and spread among them.

To prepare for future pandemics, the international research community needs to continue and further strengthen research efforts in various areas, including the following: (i) cataloging the landscape and animal reservoirs of (human-infecting) viruses through surveillance and metagenomics; (ii) development of animal models for viruses that may cause pandemics; (iii) basic research to better understand the molecular virology of such viruses; (iv) early stage vaccine development and testing in animal models; and (v) development of broad antivirals as a first line of defense. The US National Institute of Allergy and Infectious Diseases has suggested that prototype pathogens (selected from virus families that may cause pandemics) be selected for basic research and early stage development of countermeasures [29]. With reasonable resources and advanced technologies, the global community could be much better prepared for future pandemics.

## Figures and Tables

**Table 1:** Overview of pandemic viruses, PHEIC agents, and viruses causing WHO priority diseases.

Pandemic	PHEIC (Since 2005)	WHO Priority Diseases	Virus	Virus Family	Reservoir	Mode of Human Infection from Animal Reservoir	Mode of Human-to-Human Transmission	Approved Human Vaccine
Yes			Pandemic 1918 A/H1N1 influenza virus	*Orthomyxoviridae*	Wild waterfowl	Airborne or close contact with infected animals	Airborne	No
Yes			Pandemic 1957 A/H2N2 influenza virus	*Orthomyxoviridae*	Wild waterfowl	Airborne or close contact with infected animals	Airborne	Yes
Yes			Pandemic 1968 A/H3N2 influenza virus	*Orthomyxoviridae*	Wild waterfowl	Airborne or close contact with infected animals	Airborne	Yes
Yes			Pandemic 1977 A/H1N1 influenza virus	*Orthomyxoviridae*	Re-introduction of previously circulating strain	Re-introduction of previously circulating strain	Airborne	Yes
Yes	Yes		Pandemic 2009 A/H1N1 influenza virus	*Orthomyxoviridae*	Wild waterfowl/swine	Airborne or close contact with infected animals	Airborne	Yes
Yes	Yes	Yes	SARS-CoV-2	*Coronaviridae*	Bats	Airborne or close contact with infected animals	Airborne	Yes
	Yes	Yes	Ebola virus	*Filoviridae*	Bats	Contact/consumption of infected bats or intermediary hosts (such as nonhuman primates)	Direct contact with body fluids	Yes
	Yes	Yes	Zika virus	*Flaviviridae*	Arthropods	Arthropod-borne	Perinatal, sexual	No
	Yes		Polio virus	*Picornaviridae*	None	N/A	Fecal-oral	Yes
	Yes		Smallpox (variola) virus	*Poxviridae*	None	N/A	Droplets	Yes
	Yes		Monkeypox virus	*Poxviridae*	Most likely, small mammals	Direct contact with infected animals	Close, personal contact; fomites; contact with respiratory secretions	Yes
		Yes	Crimean-Congo hemorrhagic fever virus	*Nairoviridae*	Arthropods	Arthropod-borne	Direct contact with body fluids	No
		Yes	Marburg virus	*Filoviridae*	Bats	Contact/consumption of infected bats or intermediary hosts (such as nonhuman primates)	Direct contact with body fluids	No
		Yes	Lassa virus	*Arenaviridae*	Rodents	Contact with rodent urine or feces	Direct contact with body fluids	No
		Yes	MERS	*Coronaviridae*	Bats	Contact with intermediary hosts such as camels	Direct contact, airborne?	No
	Yes	Yes	SARS	*Coronaviridae*	Bats	Unknown (close contact with intermediary hosts?)	Airborne (inefficient)	No
		Yes	Nipah virus	*Paramyxoviridae*	Bats	Consumption of infected bats or pigs or virus-contaminated fruits	Respiratory secretions	No
		Yes	Rift Valley fever virus	*Phenuiviridae*	Arthropods	Close contact with infected livestock	No	No
		Yes	A virus responsible for ‘Disease X’	???	???	???	???	N/A

**Table 2:** Overview of major human respiratory viruses.

Virus Family	Genus	Representative Human Virus(es)	Representative Non-human Virus(es)	Human Vaccines	Antiviral Treatments
Orthomyxoviridae	Alphainfluenzavirus	Human influenza A viruses	Avian influenza A viruses Swine influenza A viruses Horse influenza A viruses	Yes	Neuraminidase and polymerase inhibitors
	Betainfluenzavirus	Human influenza B viruses			
Paramyxoviridae	Orthopneumovirus	Human respiratory syncytial virus	Bovine respiratory syncytial virus	None	Ribavirin, monoclonal antibody
	Respirovirus	Human parainfluenza viruses 1 & 3	Bovine respirovirus Murine respirovirus Porcine respirovirus	None	None
	Rubulavirus	Human parainfluenza viruses 2 & 4	Newcastle disease virus	None	None
	Metapneumovirus	Human metapneumovirus	Avian metapneumovirus	None	None
	Morbillivirus	Measles virus (Measles morbillivirus)	Canine distemper virus	Yes	None
	Orthorubulavirus	Mumps virus (Mumps orthorubulavirus)	Simian orthorubulavirus Porcine orthorubulavirus	Yes	None
Picornaviridae	Enterovirus	Human rhinovirus		None	None
	Aphtovirus		Bovine rhinitis A virus Equine rhinitis A virus Foot-and-mouth disease virus	None	None
Coronaviridae	Alphacoronavirus	Human coronavirus 229E, NL63	Bat coronaviruses	None	None
	Betacoronavirus	SARS-CoV SARS-CoV-2 Human coronavirus OC43, HKU1	Bat coronaviruses Murine coronaviruses	None Yes None	None Remdesivir, Paxlovid, Molnupiravir None
Adenoviridae	Mastadenovirus	Human adenovirus	Bat mastadenovirus Murine mastadenovirus Simian mastadenovirus Porcine mastadenovirus	None	None
Parvoviridae	Erythroparvovirus	Human parvovirus B18	Primate erythroparvovirus Rodent eryhtroparvovirus Ungulate eryhtroparvovirus	None	None

## Data Availability

Not applicable.
